# Supplementary material for: Resolving the full spectrum of human genome variation using Linked-Reads
Source: Genome Res. 2019 Apr;29(4):635–45. doi: 10.1101/gr.234443.118 (PMC6442396; doi:10.1101/gr.234443.118)
Supplement: Supplemental Material [file supp_gr.234443.118_Supplemental_longranger-2.2.zip › longranger-2.2.tar.bz2!/lr2.2-public/lib/stan/cmdstan/stan/doc/api/html/dist_8hpp_source.html]

Stan: src/stan/math/functions/dist.hpp Source File


|  |  |
| --- | --- |
|  | Stan  2.5.0  probability, sampling & optimization |


- Main Page
- Related Pages
- Namespaces
- Classes
- Files

- File List
- File Members

All Classes Namespaces Files Functions Variables Typedefs Enumerations Enumerator Friends Macros Pages

- src
- stan
- math
- functions

dist.hpp

Go to the documentation of this file.

1 #ifndef STAN\_\_MATH\_\_FUNCTIONS\_\_DIST\_HPP

2 #define STAN\_\_MATH\_\_FUNCTIONS\_\_DIST\_HPP

3

4 #include <vector>

5 #include <cstddef>

6 #include <cmath>

7

8 namespace stan {

9  namespace math {

10

11  inline double dist(const std::vector<double>& x, const std::vector<double>& y) {

12  using std::sqrt;

13  double result = 0;

14  for (size\_t i = 0; i < x.size(); ++i) {

15  double diff = x[i] - y[i];

16  result += diff \* diff;

17  }

18  return sqrt(result);

19  }

20

21  }

22 }

23

24 #endif

stan::agrad::sqrt

fvar< T > sqrt(const fvar< T > &x)

**Definition:** sqrt.hpp:15

stan::math::dist

double dist(const std::vector< double > &x, const std::vector< double > &y)

**Definition:** dist.hpp:11


---

[ Stan Home Page ]

*© 2011–2014,
Stan Development Team.*
